# Are juvenile offenders psychologically different from juvenile criminals? A preliminary investigation

**DOI:** 10.3389/fpsyg.2026.1704471

**Published:** 2026-05-26

**Authors:** Sanyang Liu, Peng Xiang, Haifeng Li, Dayong Xu, Jiahao Lu, Bowen Chen, Weibo Jian, Mingsen Wang, Linjie Qu

**Affiliations:** 1 School of Law, Jiangsu Normal University, Xuzhou, China; 2 Nanjing University of Finance and Economics, Nanjing, China; 3 East China University of Political Science and Law, Shanghai, China; 4 The People's Procuratorate of Xinyi, Xinyi, China; 5 The Eighth Prosecution Department, People's Procuratorate of Suzhou, Suzhou, China; 6 Koguan School of Law, Shanghai Jiaotong University, Shanghai, China; 7 Northwest University of Politics and Law, Xi’an, China; 8 The People’s Court of Minhang District, Shanghai, China

**Keywords:** belief in a just world, empathy, juvenile criminals, juvenile offenders, psychologically, self-control

## Abstract

**Objective:**

To better serve the best interests of minors, there is a growing consensus on the need for differentiated correction approaches for juvenile offenders based on their classifications. To implement these tailored interventions, it is essential to examine the psychological differences among various subgroups of juvenile offenders. Particular attention should be given to the distinctions between those undergoing correctional supervision and those serving sentences in different institutional settings. Additionally, whether it is the consensual understanding that affirms the subtle relationship between the psychological organism and environmental factors, or the legal provisions of various countries, a hypothesis seems to be implied: that juvenile offenders and juvenile criminals, who are separately housed or corrected in different environments, exhibit certain psychological differences. This study was designed to test this hypothesis.

**Method:**

To test this hypothesis, the present study utilized a cross-sectional survey design. We employed convenience sampling to administer a questionnaire to 178 juvenile offenders from specialized educational institutions in J Province, China, and to 303 juvenile criminals from local detention centers for comparison.

**Results:**

Data analysis showed the following findings: (1) juvenile offenders scored significantly lower than juvenile criminals on belief in a just world—including total score, general belief, and personal belief. (2) No significant difference was found between the two groups in self-control. (3) Regarding empathy, juvenile offenders scored significantly lower on affective empathy than juvenile criminals, but no significant differences were found in total empathy or cognitive empathy. (4) Female juvenile offenders reported lower personal belief in a just world but higher affective empathy than their male counterparts.

**Conclusion:**

Compared with juvenile criminals, juvenile offenders do not show a significantly lower risk in delinquent acts, continued follow-up on their psychological status remaining essential.

## Introduction

In today’s world, the classified treatment of delinquent juveniles reflects a legislative consensus among nations. In the United States, juveniles are categorized based on the age at which the offense occurs and the type of norm violated. This classification includes status offenders, predelinquents, general delinquent juveniles, and juvenile offenders (Chen and He, 2021). Similarly, in Germany, offenders are classified by the juvenile justice system as persons without criminal responsibility, juvenile criminals, or young adults liable under criminal law, depending on their age at the time of the offense (Zhang, 2005). In the People’s Republic of China, where this study is based, delinquent juveniles are also categorized according to age and the nature of the norm violated, into predelinquents, general delinquent juveniles, and juvenile offenders. The rationale for adopting these classified treatment measures is twofold: first, from the principle of culpability, it allows for differential treatment based on the harm caused to society; second, from a welfarist perspective, it implements correctional measures that best support the minor’s development, taking into account their individual psychosomatic characteristics (Gao, 2024).

Classified treatment aims to provide individualized rehabilitation for distinct groups of delinquent juveniles, making it essential to thoroughly understand the psychosomatic conditions of each group. This involves examining the psychological states of various types of delinquent juveniles and exploring the psychological differences among these groups. Effective psychological correctional measures are crucial for delivering favorable treatment interventions (Pandey, 2022). They provide necessary rehabilitative therapy, address psychological trauma (Rhoden et al., 2019), reduce problem behaviors and associations with negative peers, and lower recidivism rates (Sulasa and Kumar, 2024). In China, the psychological health of adolescents is particularly significant. A study found that 54.7% of guardians expressed a strong desire for family education services focused on “children’s psychological health” (Bian and Zhang, 2021). In summary, beneficial psychological correctional measures play a vital role in the resocialization of delinquent juveniles. However, to implement these measures effectively, it is crucial to first investigate the psychological differences among the various groups of delinquent juveniles.

While the psychological conditions of status offenders (Martin, 1977), predelinquents (Phillips et al., 2024; Gómez Tabares, 2025), general delinquent juveniles, and juvenile offenders (Baráth, 2023) have received scholarly attention, there is comparatively little literature examining the psychological differences among these groups. Specifically, the distinctions between juvenile offenders and juvenile criminals undergoing correctional interventions in different environments have been underexplored. In psychological research, both groups are often categorized under the broader term “delinquent juveniles” (Kolesnikov, 2022; Aazami et al., 2023; Fareed and Javed, 2025), with their differences seldom addressed. Given the recognized interaction between psychological factors and the immediate environment, it is important to acknowledge the psychological disparities that exist between these two groups.

Bronfenbrenner, the proponent of ecological systems theory in human development, argued that individuals are directly influenced by systems such as family, school, and workplace, while also being indirectly shaped by policies, resources, and societal expectations. He emphasized that dynamic environments significantly impact developing individuals, who in turn can influence their surroundings (Bronfenbrenner and Morris, 2006). He described the complex, bidirectional interactions between a human’s biopsychological makeup and their immediate environment as proximal processes (Bronfenbrenner and Morris, 1998). These processes yield effects only when they occur consistently over extended periods. The direction and magnitude of these effects can vary greatly based on the characteristics of both the environment and the individual. Additionally, the form, power, content, and direction of proximal processes are systematically influenced by individual traits, environmental features, the nature of developmental outcomes, the social conditions faced, and the historical context (Bronfenbrenner, 1994). The development of a child’s psychological organism exemplifies this principle: “The psychological development of the child is achieved through progressively more complex patterns of reciprocal activity that occur with persons to whom the child has established a strong and enduring mutual emotional attachment” (Bronfenbrenner, 1985). Furthermore, “the greater developmental impact of proximal processes among children growing up in disadvantaged or disorganized environments is expected to be manifested primarily in outcomes reflecting developmental dysfunction. In contrast, for outcomes reflecting developmental competence, proximal processes may have greater impact in more advantaged and stable environments” (Bronfenbrenner and Morris, 1998).

Erik Erikson, the proponent of the epigenetic model (Erikson, 1963), argued that every child goes through a series of developmental stages from infancy to old age. Each child must develop their own regulatory mechanisms within the dynamic interplay of their inner voice, physiological and emotional impulses, and societal influences. Throughout these stages, children learn to balance self-regulation by coping with new instincts and understanding themselves and others. For adolescents, ages 10 to 24/26, finding a balance between identity and role is confusing. While achieving a sense of belonging in their social context is especially crucial. Erikson noted, “…it is primarily the inability to settle on an occupational identity which disturbs young individuals. To keep themselves together, they temporarily overidentify with the heroes of cliques and crowds to the point of an apparently complete loss of individuality” (Erikson, 1963).

In recent years, there has been a growing scholarly focus on how environmental factors influence the development of the adolescent biopsychological organism. For instance, Hazen et al. (2008) discovered that the parental and adult values were significant negative predictors of multiple adolescent difficulties. These included peers and family relationship problems, depression, risky sexual behavior, risk-taking tendencies, academic underachievement, and substance abuse. The relationship was largely explained by the influence of these values on adolescents’ self-image. Additionally, physical illness, regardless of its visible effects on appearance, was shown to have detrimental effects on self-esteem. Colich et al. (2020) found that supportive caregiving can significantly improve adolescent mental health, even for those who faced considerable psychosocial stress in childhood. Furthermore, Turpyn et al. (2021) revealed that individual differences in physiological responses to expected social rewards, specifically activation in the ventral striatum and amygdala, moderated the effects of family conflict on adolescents’ psychological outcomes.

Juvenile offenders and juvenile criminals display significant differences, whether viewed from a biopsychological perspective or in terms of their immediate environments. Consequently, their psychological states should also vary notably. According to the Law of the People’s Republic of China on the Prevention of Juvenile Delinquency, the criteria for addressing these two groups of minors differ substantially.

Juvenile offenders are defined as those who have committed serious legal violations but have not demonstrated remorse after undergoing correctional education mandated by administrative authorities. These individuals often come from families and schools that cannot enforce effective discipline. Additionally, some juvenile offenders may have committed criminal acts but are not subject to criminal punishment due to not meeting the age of criminal responsibility, resulting in their placement in specialized educational institutions. In contrast, juvenile criminals are individuals who have committed criminal acts and have reached the age of criminal responsibility, leading to their detention in correctional facilities. In brief, juvenile offenders are typically younger than juvenile criminals, and their psychological characteristics differs considerably. Furthermore, the environments these two groups encounter during correctional interventions or incarceration are distinctly different. Specialized educational institutions for juvenile offenders tend to provide a more supportive atmosphere, while detention centers for juvenile criminals are characterized by stricter regulations, rigorous daily routines, mandatory labor, limited access to education, and increased interaction with peers viewed as more “dangerous.” Moreover, the post-release supervision of these two groups varies significantly. Juvenile offenders return directly to their families and schools after completing their intervention, without any further oversight from the state. In contrast, juvenile criminals remain subject to a range of state-imposed correctional measures upon release, including psychological assessments, vocational training, resettlement support, and designated social supervision. Thus, from the state’s perspective, juvenile offenders and juvenile criminals are seen as having different psychological states, with juvenile criminals often viewed as more psychologically impaired. Is this perception accurate from a psychological standpoint? Accordingly, we propose the following hypothesis:

*H1:* Are juvenile criminals psychologically different from juvenile offenders?

This study preliminarily selected three measurement constructs: self-control ability, empathy, and belief in a just world, to explore the psychological states of juvenile criminals and offenders. Self-control ability and empathy are commonly used as indicators of the psychological conditions of offenders and delinquent minors in both Chinese and international literature (Chaplin et al., 2021; Walters, 2025; Javakhishvili and Vazsonyi, 2022; Wallner et al., 2021; Helmut et al., 2023; Hirtenlehner et al., 2023; Zhou et al., 2018; Teng, 2018). Additionally, the relationship between belief in a just world and juvenile delinquency has also received significant attention (Hofer and Spengler, 2020; Fang, 2017). This emphasis suggests a consensus on the relevance of these constructs.

(1) Belief in a just world is the conviction that individuals inhabit a fair environment where outcomes are equitable, efforts are rewarded, and sustained commitment to a goal is valuable (Lerner and Miller, 1978). Research shows that, among juvenile offenders, a strong belief in a just world correlates with fewer disciplinary infractions, mediated by justice motivation (Otto and Dalbert, 2005). For pre-offending juveniles, a general belief in a just world is significantly negatively correlated with aggressive behaviors such as physical aggression, anger, and hostility. Furthermore, personal belief in a just world is a significant negative predictor of aggressive behavior (Ren, 2021). (2) Self-control ability is defined as the capacity to suppress inappropriate emotions and behaviors and replace them with suitable actions (Casey, 2015). Individuals with low self-control are more likely to engage in a variety of adverse behaviors, particularly antisocial and criminal activities (Walters, 2016). (3) Psychological research indicates that empathy development influences offending behavior among juvenile criminals (Narvey et al., 2021). Empathy is the cognitive ability to understand and share the emotions of others (Jolliffe and Farrington, 2004). Low empathy is often linked to aggressive behavior and criminality, increasing the likelihood of offending, especially violent offenses (Jolliffe and Farrington, 2007). Conversely, high levels of empathy are associated with reduced violence and aggression (Chialant et al., 2016). Thus, in addition to the indicators of belief in a just world (1) and self-control ability (2), this study also incorporates empathy (3) as a measure of the psychological state of minors. Consequently, the previously proposed hypothesis H1 can be refined and restated as H2:

*H2:* Compared to juvenile criminals, juvenile offenders are psychologically different in belief in a just world, self-control, and empathy.

### The current study

This study utilized a quantitative analysis approach. First, we compared the scores of juvenile offenders and juvenile criminals on measures of belief in a just world, self-control ability, and empathy, using a scale designed to assess tendencies related to violent offending. Second, after controlling for demographic variables such as parental education level and family socioeconomic status, we investigated the relationships between these three psychological attributes and age among juvenile criminals. Ultimately, this study aimed to address the following research questions:

- Do juvenile offenders differ from juvenile criminals in their belief in a just world and other key psychological variables?
- What interrelationships exist among belief in a just world and other core variables among juvenile offenders?
- How can specialized educational institutions be transformed into environmental factors that promote healthy and positive psychological effects on juvenile offenders?

## Method

### Participants

This study utilized a cross-sectional survey design and convenience sampling to gather a total of 481 valid responses from J Province, China, which included 178 juvenile offenders and 303 juvenile criminals. J Province, located in eastern China, is one of the nation’s most economically developed regions, with a per capita disposable income (PCDI) of ¥32,414 to ¥66,173 in 2024, compared to the national PCDI of ¥23,119 to ¥54,188. Data from the juvenile offenders were collected at a specialized educational institution in X City, J Province, resulting in 178 valid questionnaires (161 males and 17 females). The ages of these juvenile offenders ranged from 10 to 18 years, with a mean age of 15.41 years. In terms of family residence, 39.33% came from urban areas, 52.81% from rural areas, and 1.20% did not report their residence. Regarding parental educational attainment, a significant proportion of participants indicated that their fathers (58.43%) or mothers (48.31%) had completed secondary education (junior or senior high school).

For comparative purposes, this study collected data from a sample of juvenile criminals as a reference group. To respect the cultural characteristics and regional diversity within J Province, we recruited 303 valid respondents (288 males and 15 females) from detention centers in S City and X City. All participants had been adjudicated and sentenced for violations of the Criminal Law of the People’s Republic of China, with ages ranging from 13 to 18 years (M = 16.65 years). In terms of family residence, 27.06% were from urban areas, 65.68% from rural areas, and 7.26% did not report their residence. Regarding parental education, a significant number of respondents indicated that their fathers (63.37%) or mothers (59.41%) had completed secondary education (junior or senior high school). The primary offenses committed by these respondents included rape (36.96%), theft (21.78%), and robbery (11.55%). Most respondents received principal sentences of fixed-term imprisonment ranging from one to two years (22.77%), three years or more (19.14%), or criminal detention (11.88%).

This study received rigorous ethical approval from the Institutional Review Board of the first author’s institution (Approval No. H2024-14), ensuring compliance with established academic ethical standards. Additionally, the research adhered to the Declaration of Helsinki. Throughout the survey process, participants were given detailed information about the study’s purpose, procedures, potential risks, and their rights, including the right to withdraw at any time. Informed consent was obtained accordingly. All personally identifiable information related to minors was either fully anonymized or not recorded.

### Measures

#### Belief in a just world scale

This study utilized the Chinese version of the Belief in a Just World Scale, originally developed by Lipkus et al. (1996). They identified two dimensions of belief in a just world and created a two-dimensional scale to evaluate this concept. The scale consists of two subscales: Personal Belief in a Just World (PBJW), which measures how fair individuals perceive the world to be towards themselves, and General Belief in a Just World (GBJW), which assesses their perception of fairness towards others. The scale contains 13 items, with 7 items dedicated to PBJW and 6 items to GBJW. Participants rate the items on a 6-point Likert scale, ranging from 1 (strongly disagree) to 6 (strongly agree). Su et al. (2012) translated and revised the scale, confirming its satisfactory reliability and validity among Chinese participants. In the current study, Cronbach’s *α* coefficients for the overall Belief in a Just World Scale, the GBJW subscale, and the PBJW subscale were 0.93, 0.86, and 0.90, respectively.

#### Self-control ability questionnaire

This questionnaire, created by Chinese scholars Wang and Lu, aims to evaluate self-control ability in minors aged 12 to 18 years. It measures self-control across three dimensions: emotional self-control, behavioral self-control, and cognitive self-control. The scale has a split-half reliability of 0.856 (Wang and Lu, 2004). Comprising 36 items, 10 positively worded and 26 negatively worded, each item is rated on a 5-point Likert scale from 1 (strongly disagree) to 5 (strongly agree), with higher scores reflecting greater self-control ability. In this study, the Cronbach’s *α* for the Self-Control Ability Questionnaire was 0.90.

#### Basic empathy scale

The present study utilized the Basic Empathy Scale (BES; Darrick and David, 2006) to measure empathy. The BES includes two dimensions: affective empathy and cognitive empathy. It consists of 20 items, comprising 8 reverse-scored items and 12 positively worded items, each rated on a 5-point Likert scale from 1 (strongly disagree) to 5 (strongly agree). Higher scores reflect greater empathy. Li et al. (2011) evaluated the theoretical factor structure, reliability, and validity of the BES in a sample of Chinese adolescents, confirming that the scale meets psychometric standards (α = 0.78). In the current study, the Cronbach’s α coefficients for the overall BES, the cognitive empathy subscale, and the affective empathy subscale were 0.72, 0.77, and 0.73, respectively.

### Plan of analysis

In this study, all analyses were performed using SPSS (Statistical Product and Service Solutions) version 27.0. Prior to data analysis, scores were computed for the Belief in a Just World Scale (BJWS), the Self-Control Questionnaire, and the Basic Empathy Scale (BES). Following these preparatory steps, an analysis of covariance (ANCOVA) was first conducted to examine differences between juvenile criminals and juvenile offenders in BJWS scores, self-control scores, and BES scores. Subsequently, one-way ANOVA or independent-samples t-tests were used to assess the effects of demographic variables among juvenile offenders on their belief in a just world, self-control, and empathy. Statistical significance was set at *p* < 0.05 (two-tailed).

For study variables with missing data (belief in a just world, empathy and its subdimensions, self-control, and parental education level; missing rates ranging from 1.66 to 10.19%), Little’s test for missing completely at random (MCAR) was performed. The results strongly supported that the data were MCAR (χ^2^(132) = 41.03, *p* > 0.05). Nevertheless, to maximize statistical power, obtain more accurate estimates, and adhere to current methodological standards of rigor, multiple imputation by chained equations (MICE) was employed to handle missing values. The imputation model included all analytic variables to preserve relationships among them. Using the Multiple Imputation module in SPSS 27.0, all study variables were entered into the imputation model, and appropriate methods were specified based on variable type (linear regression for continuous variables, logistic regression for categorical variables). After generating m = 50 complete datasets, all subsequent statistical analyses were conducted on this multiply imputed data.

Normality of the primary variables was assessed using the Shapiro–Wilk test. The normality test results across the 50 imputed datasets indicated that for belief in a just world and its subdimensions (general belief in a just world and personal belief in a just world), as well as for self-control, the Shapiro–Wilk statistics ranged from 0.98 to 0.99, and Q-Q plots showed that the data points closely followed the diagonal line, suggesting approximate normality. These variables were therefore subjected to parametric statistical analyses. In contrast, for self-control and its subdimensions (cognitive empathy and affective empathy), the Shapiro–Wilk statistics ranged from 0.95 to 0.98, and Q-Q plots revealed systematic deviations; moreover, none of the attempted transformations substantially improved normality. Consequently, non-parametric tests were adopted for subsequent data processing for these variables.

## Results

For variables that met the normality assumption (belief in a just world and its subdimensions, self-control), a parametric analysis of covariance (ANCOVA) was conducted. The ANCOVA included grouping (juvenile criminals vs. juvenile offenders) and gender as fixed factors, with centered age, age-squared, father’s education level, and mother’s education level as covariates. The group × centered age interaction was also examined to determine whether the effect of age on the dependent variables differed between groups. All analyses were based on multiply imputed data (m = 50), and results were pooled following Rubin’s rules (Table 1).

**Table 1:** Between-group comparisons of key outcome variables (pooled across 50 imputations).

Dependent variable	Grouping effect	Gender effect	Age effect	Group × Age interaction	Father’s education level	Mother’s education level
Belief in a just world	*F* = 60.20***	*F* = 2.35	*F* = 1.96	*F* = 0.86	*F* = 0.14	*F* = 0.45
General belief in a just world	*F* = 58.08***	*F* = 0.14	*F* = 3.36	*F* = 0.79	*F* = 0.24	*F* = 1.29
Personal belief in a just world	*F* = 44.89***	*F* = 4.89*	*F* = 0.72	*F* = 0.67	*F* = 0.05	*F* = 0.04
Self-control	*F* = 0.14	*F* = 0.01	*F* = 0.00	*F* = 0.01	*F* = 0.47	*F* = 1.91
Empathy	*F* = 0.76	*F* = 3.80	*F* = 0.66	*F* = 0.69	*F* = 0.55	*F* = 0.36
Affective empathy	*F* = 4.48*	*F* = 6.45**	*F* = 0.68	*F* = 2.56	*F* = 0.01	*F* = 0.01
Cognitive empathy	*F* = 1.08	*F* = 0.01	F = 0.84	*F* = 0.79	*F* = 0.06	*F* = 0.24

* *p* < 0.05, ** *p* < 0.01, * *p* < 0.001. *F*-values without superscripts indicate *p* ≥ 0.05.

Regarding belief in a just world, juvenile criminals scored significantly higher than juvenile offenders on the total scale (*B* = 9.58, *SE* = 1.24, *t* = 7.76, *p* < 0.001, 95% CI [7.16, 12.01]) as well as on its two subdimensions—general belief in a just world (*B* = 4.56, *SE* = 0.60, *t* = 7.62, *p* < 0 0.001, 95% CI [3.39, 5.73]) and personal belief in a just world (*B* = 5.03, *SE* = 0.75, *t* = 6.70, *p* < 0.001, 95% CI [3.56, 6.50]), all *p* < 0.001. Gender was significant only for personal belief in a just world (*B* = 2.59, *SE* = 1.17, *t* = 2.21, *p* = 0.03, 95% CI [0.30, 4.89]), indicating that males had significantly higher scores than females. Parental education levels and age had no significant effects on any of the belief in a just world variables (*p* > 0.05). The group × age interaction was not significant (*p* > 0.05), suggesting that the pattern of age effects on belief in a just world was consistent across the two groups.

For self-control, after controlling for age (linear and nonlinear), gender, and parental education levels, no significant difference was found between juvenile criminals and juvenile offenders (*t* = −0.37, *p* = 0.72, *B* = −0.96, *SE* = 2.61, 95% CI[−6.08, 4.17]). The main effects of all demographic covariates (age: linear *p* = 0.99, nonlinear *p* = 0.56; gender *p* = 0.94; parental education levels *p* > 0.05) were not significant (*p* > 0.05). The age × group interaction was also not significant (*p* = 0.94).

Because empathy and its subdimensions did not satisfy the normality assumption (Shapiro–Wilk = 0.95–0.98), an aligned rank ANCOVA was performed as a robustness check. The procedure involved the following steps: first, the dependent variables and covariates (centered age, age-squared, parental education levels) were rank-transformed; second, the ranks of the dependent variables were regressed on the ranks of the covariates, and the unstandardized residuals were saved; finally, a factorial ANOVA was conducted on the residuals, with group and gender as fixed factors, while also testing the group × centered age interaction.

For total empathy, the main effect of group was not significant (*F* = 0.87, *p* = 0.39), the main effect of gender was marginally significant (*F* = 3.80, *p* = 0.05), and the group × age interaction was not significant (*F* = 0.69, *p* = 0.42). For cognitive empathy, none of the effects reached significance (group: *F* = 1.08, *p* = 0.30; gender: *F* = 0.01, *p* = 0.91; age: *F* = 0.84, *p* = 0.36; group × age: *F* = 0.79, *p* = 0.38; all *p* > 0.05). For affective empathy, significant group differences emerged: juvenile criminals scored significantly higher than juvenile offenders (*F* = 3.5–5.4, *p* = 0.02–0.05), and females scored significantly higher than males (*F* = 5.5–7.4, *p* = 0.007–0.02). The group × age interaction was marginally significant (*F* = 2.0–3.1, *p* = 0.07–0.10).

## Discussion

### Belief in a just world

The present study revealed several key findings: (1) Both juvenile offenders and juvenile criminals demonstrated a firmer personal belief in a just world compared to their general belief in a just world, aligning with Dalbert’s (1999) research. This phenomenon may be linked to self-serving bias (Dalbert, 1999), the development of abstract reasoning skills in adolescents (Dalbert and Radant, 2004), or the impact of friendship quality and online social interactions (Tian et al., 2019). (2) Juvenile offenders have a significantly lower general belief in a just world than juvenile criminals. We hypothesize that this discrepancy may stem from differences in childhood experiences and parenting styles. Jin and Yang (2025) established that childhood adversity (stressful environments during childhood) and perceptions of injustice (perceived discrimination) predict adolescents’ belief in a just world over time. Additionally, Sun et al. (2024) found a significant negative correlation between parental psychological control among Chinese parents and their children’s belief in a just world. (3) Among juvenile offenders, belief in a just world was significantly positively correlated with empathy, supporting findings from Liu (2022) and Xu et al. (2024). (4) Male juvenile offenders reported a significantly firmer personal belief in a just world than their female counterparts. This result contrasts with that of Liu et al. (2008), which indicated that female minors scored higher than males on personal belief in a just world. This might be related to the group differences between ordinary female minors and female juvenile offenders.

### Self-control

Gottfredson and Hirschi (1990) introduced the stability theory, which posits that self-control develops over the life course. This theory suggests that while individuals may enhance their self-control as they age, differences in self-control are largely established within the first decade of life and tend to remain constant thereafter. They argue that effective parental socialization is the primary source of self-control, with other social environmental factors that have minimal impact. However, research indicates that self-control can be more malleable than suggested. Studies have shown considerable variability in self-control among individuals and significant differences in developmental trajectories among adolescents aged 12 to 16 years (Meldrum et al., 2012). Additionally, while many studies have found a positive correlation between effective parenting and adolescent self-control (Burt et al., 2006; Hay and Forrest, 2006; Nofziger, 2008; Vazsonyi and Huang, 2010), emerging research has identified other influential factors overlooked by Gottfredson and Hirschi. These include genetic and biological variables (Wright and Beaver, 2005), neighborhood environments (Gibson et al., 2010), school experiences (Turner et al., 2005), and peer associations (Hay and Meldrum, 2015; Meldrum and Hay, 2012; Meldrum et al., 2012). The present study found no statistically significant difference in self-control between juvenile offenders and juvenile criminals. This outcome appears to support Gottfredson and Hirschi’s stability theory, particularly considering the differing correctional environments, peer groups, and parental educational backgrounds of the two populations. However, it is important to acknowledge the study’s limitations. The inability to examine genetic and biological variables, parenting styles, neighborhood conditions, or school experiences means that these potentially influential factors cannot be dismissed as having a more significant impact on self-control in these groups than currently understood.

### Empathy

The present study revealed the following findings: (1) Juvenile criminals showed no significant differences from juvenile offenders in total empathy score or cognitive empathy; however, their affective empathy was significantly lower than that of juvenile offenders (Huang and Su, 2010). One possible explanation for this pattern is that juvenile criminals, having been raised in complex and adverse social environments, may develop heightened emotional sensitivity as a means of coping with potential social threats. Research has shown that individuals who have experienced trauma or live in high-risk settings may exhibit adaptive enhancements in emotional awareness and empathic responding (Toplu and Çakmak, 2024). Another explanation is that the differences between the two groups may reflect distinct emotion regulation strategies. Although juvenile criminals display higher affective empathy, they may lack the corresponding cognitive regulatory abilities, such that their affective empathy fails to translate into prosocial behavior and may even co-occur with aggression in certain contexts. This interpretation aligns with the finding of no significant group difference in cognitive empathy. (2) Male juvenile offenders demonstrated significantly lower levels of affective empathy compared to their female counterparts. Aligning with previous research that indicates adolescent girls exhibit higher empathy, both cognitive and affective than boys (Christov-Moore et al., 2014). Christov-Moore found that females exhibit more advanced brain structure development compared to males, a finding that may be related to the aforementioned differences. Furthermore, the greater cultural emphasis on empathy and prosocial behavior in females, which reflects a sense of “conscious mentalization,” is also thought to be associated with gender differences in human affective empathy.

With regard to the study’s conclusions, the present findings partially corroborate previous insights concerning the association between the psychological organism and environmental factors. Juvenile offenders and juvenile criminals are placed in distinct correctional settings, and corresponding differences do exist in their psychological states. Nevertheless, this observation calls for further empirical validation. On the one hand, factors such as family residence and parental education level showed no association with the psychological variables under investigation. On the other hand, the potential confounding effect of preexisting group differences on the results cannot be completely ruled out.

Integrating the current findings with those of previous research, we recommend that:

1. More attention should be paid to the psychological status of juvenile offenders. Compared with juvenile criminals, juvenile offenders scored lower on belief in a just world and affective empathy, whereas they did not differ significantly in self-control or total empathy. Given the established correlations between these core variables and aggressive or other deviant behaviors, the risk of these individuals engaging in delinquent acts cannot be discounted. In the current Chinese context, once juvenile offenders leave specialized education institutions after completing six to twelve months of correctional education, they no longer receive systematic follow-up—a practice that calls for critical reflection. Longitudinal research on their psychological status should be actively pursued.
2. Family parenting styles of delinquent offenders merit attention. The present study found no correlation between parental education level and adolescents’ belief in a just world, self-control, or empathy. In contrast, prior research indicates that parenting styles directly and positively affect children’s self-control. Specifically, authoritative parenting is linked to higher self-control in children (Papadopoulos, 2021). Parenting styles also predict adolescents’ personal belief in a just world (Wang et al., 2024) and empathy (Ramadhanti et al., 2023). Thus, higher parental education does not guarantee better psychological outcomes in adolescents—an important insight for Chinese families. Nevertheless, the relationship between juvenile offenders’ psychological states and their family parenting styles requires further empirical investigation.
3. Priority should be given to fostering a high-quality classroom climate within specialized education institutions. As noted above, compared with the detention centers where juvenile criminals are held, specialized education institutions offer the distinct advantage of providing juvenile offenders with more classroom time. Previous research indicates that school-related factors and parental supervision jointly predict the development of self-control in adolescents. Moreover, a positive school climate can buffer the negative effects of poor parental supervision on adolescent self-control (Dou et al., 2022). Conversely, a low-quality classroom climate may exacerbate the adverse impact of poor parental supervision on adolescent self-control (Fisher and Brown, 2018). Additional studies have shown that teachers’ use of positive management techniques is positively associated with students’ high levels of personal belief in a just world, even when the overall school climate is suboptimal.
4. Particular attention should be devoted to the psychological status of female juvenile offenders. Ma et al. (2022) found that adolescents’ belief in a just world negatively influences their problem behaviors through the mediating effects of sense of security and cognitive reappraisal. Notably, the effect of belief in a just world on problem behaviors is weaker among adolescent females than among males, implying that even when females and males share the same level of belief in a just world, females exhibit fewer problem behaviors. However, the present study revealed that female juvenile offenders have significantly lower personal belief in a just world than their male counterparts, indicating an extremely low level of such belief within the female subgroup. Therefore, despite the smaller number of female juvenile offenders, their psychological status is a major cause for concern.

### Limitation and future studies

While the current study reached some conclusions about the psychological states of juvenile offenders compared to juvenile criminals, several limitations should be acknowledged.

This study utilized a cross-sectional design, collecting data at a single time point. Participants’ responses may have been affected by their temporary emotional states, situational factors, or fatigue at the time of assessment, which could introduce measurement error. Although measures like anonymization and reverse-scored items were implemented to address this issue, these influences cannot be eliminated. Future research should adopt intensive longitudinal designs, such as diary methods or experience sampling, to repeatedly measure core variables across various daily contexts. This approach would effectively separate stable traits from state fluctuations, improve measurement precision, and provide insights into short-term interactions among variables.

Second, this study’s sample was primarily drawn from J Province in China, raising concerns about sample representativeness. J Province is one of the most socioeconomically developed regions in the country, which means the study may not adequately represent minors from economically disadvantaged areas. Future research should utilize nationwide sampling to better assess the robustness of these findings.

Third, all data in this study were collected through self-report measures, which may be influenced by social desirability bias. To gain a more comprehensive understanding of the core constructs, future research should utilize multiple data sources and methodologies. For instance, when measuring empathy, researchers could include not only self-report questionnaires but also peer reports, behavioral observations, and objective performance indicators.

Moreover, given the inherently preliminary and exploratory nature of this study, it was not feasible to predetermine an exhaustive set of variables that would fully capture the psychological differences between the two groups. As a result, certain sample characteristics, such as socioeconomic status, were not collected. In addition, considerable difficulties were encountered in obtaining data from the participants, leading to a limited sample size and an uneven age distribution. Furthermore, due to stringent privacy protection measures, many questionnaire items were intentionally designed as optional. These factors inevitably limit the generalizability of the findings.

Finally, this study was conducted in the People’s Republic of China. Due to differences in historical and cultural contexts, levels of economic development, and geographical environments across countries, there may be significant variations in the developmental profiles of minors. Therefore, the applicability of this study’s conclusions to other nations is yet to be established.

## Conclusion

We found that juvenile offenders scored significantly lower than juvenile criminals on belief in a just world—including total score, general belief, and personal belief. No significant difference emerged between the two groups in self-control. Regarding empathy, juvenile offenders scored significantly lower on affective empathy than juvenile criminals, but no significant differences were found in total empathy or cognitive empathy. Moreover, female juvenile offenders reported lower personal belief in a just world but higher affective empathy than their male counterparts. Thus, as an initial exploration of the psychological profile of juvenile offenders, this group appears to be at no less risk of engaging in aggressive or other delinquent acts than juvenile criminals, warranting greater attention.

## Data Availability

The original contributions presented in the study are included in the article/supplementary material, further inquiries can be directed to the corresponding authors.

## References

[ref1] Aazami A. Valek R. Ponce A. N. Zare H. (2023). Risk and protective factors and interventions for reducing juvenile delinquency: a systematic review. Soc. Sci. 12:474. doi: 10.3390/socsci12090474

[ref2] Baráth N. E. (2023). Trends in juvenile delinquency from a criminal psychology and criminology perspective. Magyar Rendészet 23, 125–139. doi: 10.32577/mr.2023.1.8

[ref3] Bian Y. F. Zhang X. Y. (2021). The family education guidance service system in the new era: connotation, characteristics, and construction strategies. China Educ. Technol. 1, 20–25.

[ref5] Bronfenbrenner U. (1985). “The future of childhood,” in Children: Needs and rights, ed. Greaney V. (New York: Irvington), 167–186.

[ref6] Bronfenbrenner U. (1994). A new head start for headstart. New Horiz. Educ. 91, 57–70.

[ref7] Bronfenbrenner U. Morris P. A. (1998). The ecology of developmental processes. In Damon W. (Series Ed.) & Lerner R. M. (Vol. Ed.), Handbook of Child Psychology: Vol. 1. Theoretical Models of Human Development (5th ed., pp. 993–1028). New York: Wiley.

[ref8] Bronfenbrenner U. Morris P. A. (2006). “The bioe-cological model of human development,” in Handbook of Child psychology: Theoretical Models of Human Development, eds. Lerner R. M. Damon W. (Hoboken: Wiley), 793–828.

[ref9002] Burt C. H. Simons R. L. Simons L. G. (2006). A longitudinal test of the effects of parenting and the stability of self ‐ control: negative evidence for the general theory of crime. Criminology, 44, 353–396. doi: 10.1111/j.1745-9125.2006.00052.x

[ref9] Casey B. J. (2015). Beyond simple models of self-control to circuit-based accounts of adolescent behavior. Annu. Rev. Psychol. 66, 295–319. doi: 10.1146/annurev-psych-010814-015156, 25089362

[ref10] Chaplin T. M. Turpyn C. C. Fischer S. Martelli A. M. Ross C. E. Leichtweis R. N. . (2021). Parenting-focused mindfulness intervention reduces stress and improves parenting in highly stressed mothers of adolescents. Mindfulness 12, 450–462. doi: 10.1007/s12671-018-1026-9, 33737987 PMC7962755

[ref11] Chen L. He X. (2021). Research on the dual-track juvenile justice model. Chongqing Soc. Sci. 7, 105–117. doi: 10.19631/j.cnki.css.2021.007.009

[ref12] Chialant D. Edersheim J. Price B. H. (2016). The dialectic between empathy and violence: an opportunity for intervention? J. Neuropsychiatry Clin. Neurosci. 28, 273–285. doi: 10.1176/appi.neuropsych.15080207, 26900734

[ref13] Christov-Moore L. Simpson E. A. Coudé G. Grigaityte K. Iacoboni M. Ferrari P. F. (2014). Empathy: gender effects in brain and behavior. Neurosci. Biobehav. Rev. 46, 604–627. doi: 10.1016/j.neubiorev.2014.09.001, 25236781 PMC5110041

[ref14] Colich N. L. Rosen M. L. Williams E. S. McLaughlin K. A. (2020). Biological aging in childhood and adolescence following experiences of threat and deprivation: a systematic review and meta-analysis. Psychol. Bull. 146, 721–764. doi: 10.1037/bul0000270, 32744840 PMC7484378

[ref9005] Dalbert C. (1999). The world is more just for me than generally: About the personal belief in a just world scale’s validity. Social justice research, 12, 79–98. doi: 10.1023/A:1022091609047

[ref15] Dalbert C. Radant M. (2004). “Parenting and young adolescents' belief in a just world,” in The Justice Motive in Adolescence and Young Adulthood, 1st Edn. 11–25. London: Routledge. doi: 10.4324/9780203575802-10

[ref9004] Darrick J. David P. F. (2006). Development and validation of the Basic Empathy Scale. Journal of adolescence, 29, 589–611. doi: 10.1016/j.adolescence.2005.08.010 16198409

[ref16] Dou K. Wang L. X. Cheng D. L. Li Y. Y. Zhang M. C. (2022). Longitudinal association between poor parental supervision and risk-taking behavior: the role of self-control and school climate. J. Adolesc. 94, 525–537. doi: 10.1002/jad.12043, 35355292

[ref17] Erikson E. H. (1963). Childhood and Society, vol. 445. Norton: Bloomsbury Publishing.

[ref18] Fang C. (2017). The Relationship between Belief in a Just World and Depression among Juvenile Delinquents: The Multiple Mediating Roles of Self-Esteem and Resilience (Master's thesis). Xuzhou: Jiangsu Normal University.

[ref19] Fareed P. Javed S. (2025). Self-reported delinquent behavior among juvenile offenders and non-offenders of Sukkur, Sindh, Pakistan. Policy J. Soc. Sci. Rev. 3, 338–349.

[ref21] Fisher J. H. Brown J. L. (2018). A prospective, longitudinal examination of the influence of childhood home and school contexts on psychopathic characteristics in adolescence. J. Youth Adolesc. 47, 2041–2059. doi: 10.1007/s10964-018-0861-2, 29808318

[ref22] Gao W. (2024). On the basic principles of the graded intervention system for juvenile delinquency. J. Chin. Youth Soc. Sci. 43, 110–124. doi: 10.16034/j.cnki.10-1318/c.2024.06.012

[ref9001] Gibson C. L. Sullivan C. J. Jones S. Piquero A. R. (2010). “Does it take a village?” Assessing neighborhood influences on children’s self-control. Journal of Research in Crime and Delinquency, 47, 31–62. doi: 10.1177/0022427809348903

[ref23] Gómez Tabares A. S. (2025). Effect of adverse childhood experiences on antisocial behavior in juvenile offenders: mediation by dark personality traits and moral disengagement. J. Aggress. Maltreat. Trauma 35, 110–130. doi: 10.1080/10926771.2025.2558774

[ref24] Gottfredson M. R. Hirschi T. (1990). A General Theory of Crime. Palo Alto: Stanford University Press.

[ref9003] Hay C. Forrest W. (2006). The development of self-control: Examining self-control theory’s stability thesis. Criminology, 44, 739–774. doi: 10.1111/j.1745-9125.2006.00062.x

[ref25] Hay C. Meldrum R. (2015). Self-Control and Crime Over the Life Course. Sage Publications.

[ref26] Hazen E. Schlozman S. Beresin E. (2008). Adolescent psychological development: a review. Pediatr. Rev. 29, 161–168. doi: 10.1542/pir.29-5-161 18450837

[ref9011] Helmut H. Neema T.-B. Dirk B. Dagmar S. (2023). Does empathy attenuate the criminogenic effect of low self-control in late life?. International Journal of Comparative and Applied Criminal Justice, 47, 57–77. doi: 10.1080/01924036.2021.1955219

[ref27] Hirtenlehner H. Trivedi-Bateman N. Baier D. Strohmeier D. (2023). Does empathy attenuate the criminogenic effect of low self-control in late life? Int. J. Compar. Appl. Crim. Just. 47, 57–77. doi: 10.1080/01924036.2021.1955219

[ref28] Hofer J. Spengler B. (2020). How negative parenting might hamper identity development: spontaneous aggressiveness and personal belief in a just world. Self Identity 19, 117–139. doi: 10.1080/15298868.2018.1541026

[ref29] Huang L. Su Y. (2010). Cognitive regulation and emotion sharing processes in empathy and their relationship. J. Southw. Univ. Soc. Edn. 6, 13–19.

[ref30] Javakhishvili M. Vazsonyi A. T. (2022). Empathy, self-control, callous-unemotionality, and delinquency: unique and shared developmental antecedents. Child Psychiatr. Hum. Dev. 53, 389–402. doi: 10.1007/s10578-021-01137-2, 33580480

[ref31] Jin Y. Yang Y. (2025). How childhood shapes adolescents' belief in justice: a longitudinal study examining the link between childhood stressful environment and belief in a just world. J. Pers. 94, 252–263. doi: 10.1111/jopy.13028, 40350595

[ref32] Jolliffe D. Farrington D. P. (2004). Empathy and offending: a systematic review and meta-analysis. Aggress. Violent Behav. 9, 441–476. doi: 10.1016/j.avb.2003.03.001

[ref33] Jolliffe D. Farrington D. P. (2007). Examining the relationship between low empathy and self-reported offending. Leg. Criminol. Psychol. 12, 265–286. doi: 10.1348/135532506x147413

[ref35] Kolesnikov R. F. (2022). The current state of juvenile delinquency. Penitentiary Sci. 16, 47–56. doi: 10.46741/2686-9764.2022.57.1.005

[ref36] Lerner M. J. Miller D. T. (1978). Just world research and the attribution process: looking back and ahead. Psychol. Bull. 85, 1030–1051. doi: 10.1037/0033-2909.85.5.1030

[ref37] Li C. Yue L. Jie L. Jie Z. (2011). Initial revision of the basic empathy scale in a Chinese adolescent population. Chin. J. Clin. Psychol. 19, 163–166. doi: 10.16128/j.cnki.1005-3611.2011.02.003

[ref38] Lipkus I. M. Dalbert C. Siegler I. C. (1996). The importance of distinguishing the belief in a just world for self versus for others: implications for psychological well-being. Pers. Soc. Psychol. Bull. 22, 666–677. doi: 10.1177/0146167296227002

[ref9012] Liu W. (2022). The relationship between bystander belief in a just world, empathy, and cyberbullying intervention intention: The moderating role of social relationships (Master’s thesis). Beijing Forestry University. doi: 10.26949/d.cnki.gblyu.2022.001398

[ref40] Liu C. J. Yin X. F. Zhao R. (2008). A study on the developmental characteristics of adolescents' belief in a just world. J. Shenyang Norm. Univ. 32, 138–141. doi: 10.19496/j.cnki.ssxb.2008.06.036

[ref41] Ma M. Chen X. Lin Y. Zhang B. Bi Y. (2022). How does belief in a just world correlate with conduct problems in adolescents? The intervening roles of security, cognitive reappraisal and gender. Child. Youth Serv. Rev. 137:106432. doi: 10.1016/j.childyouth.2022.106432

[ref42] Martin C. A. (1977). Status offenders and the juvenile justice system: where do they belong. Juv. Just. 28, 7–17. doi: 10.1111/j.1755-6988.1977.tb01132.x

[ref9008] Meldrum R. C. Hay C. (2012). Do peers matter in the development of self-control? Evidence from a longitudinal study of youth. Journal of youth and adolescence, 41, 691–703. doi: 10.1007/s10964-011-9692-0 21735138

[ref43] Meldrum R. C. Young J. T. Weerman F. M. (2012). Changes in self-control during adolescence: investigating the influence of the adolescent peer network. J. Crim. Justice 40, 452–462. doi: 10.1016/j.jcrimjus.2012.07.002

[ref46] Narvey C. Yang J. Wolff K. T. Baglivio M. Piquero A. R. (2021). The interrelationship between empathy and adverse childhood experiences and their impact on juvenile recidivism. Youth Violence Juv. Justice 19, 45–67. doi: 10.1177/1541204020939647

[ref9009] Nofziger S. (2008). The “cause” of low self-control: The influence of maternal self-control. Journal of Research in Crime and Delinquency, 45, 191–224. doi: 10.1177/0022427807313708

[ref47] Otto K. Dalbert C. (2005). Belief in a just world and its functions for young prisoners. J. Res. Pers. 39, 559–573. doi: 10.1016/j.jrp.2005.01.004

[ref48] Pandey M. P. (2022). Applying Vipassanā meditation as a jail reform technique: a case of Nakkhu prison, Lalitpur, Nepal. J. Law Soc. 5, 242–250. doi: 10.11648/J.IJLS.20220503.11

[ref49] Papadopoulos D. (2021). Parenting the exceptional social-emotional needs of gifted and talented children: what do we know? Children (Basel) 8:953. doi: 10.3390/children8110953, 34828666 PMC8624036

[ref50] Phillips E. L. Wolf M. M. Fixsen D. L. Bailey J. S. (2024). “The achievement place model: a community-based, family-style, behavior modification program for predelinquents,” in Analysis of Delinquency and Aggression, 1st Edn. London: Routledge. 171–202. doi: 10.4324/9781003487630-9

[ref51] Ramadhanti A. Putra R. P. Rizky D. A. (2023). Does parenting style affect adolescent empathy? A study on high school students. Jurnal Ilm. Ilmu Terap. Univ. Jambi 7, 38–47. doi: 10.22437/jiituj.v7i1.26627

[ref52] Ren H. (2021). The Relationship between Personality Traits and Aggressive Behavior in Primary School Students: The Moderating Role of Belief in a Just World. Master Dissertation. Liaocheng: Liaocheng University. doi: 10.27214/d.cnki.glcsu.2021.000271

[ref53] Rhoden M. A. Macgowan M. J. Huang H. (2019). A systematic review of psychological trauma interventions for juvenile offenders. Res. Soc. Work Pract. 29, 892–909. doi: 10.1177/1049731518806578

[ref54] Su Z. Zhang D. Wang X. (2012). Revision of the belief scale for a just world and its reliability and validity study in college students. Chin. J. Behav. Med. Brain Sci. 21, 561–563. doi: 10.3760/cma.j.issn.1674-6554.2012.06.026

[ref55] Sulasa T. J. Kumar R. (2024). Role of modern Technology for Rehabilitation in mitigation of juvenile recidivism: a critical appraisal. Pak. J. Life Soc. Sci. 22, 11012–11018. doi: 10.57239/PJLSS-2024-22.2.00833

[ref56] Sun P. Yao X. Yuan M. Kou Y. (2024). Development of beliefs in a just world among Chinese early adolescents and the predictive role of family factors: a three-wave longitudinal study. J. Pers. 92, 1571–1586. doi: 10.1111/jopy.12912, 38111291

[ref57] Teng H. C. (2018). Research on Psychological Assessment of Minors Involved in Crime (Doctoral Dissertation). Doctoral dissertation, Yantai: Ludong University. doi: 10.27216/d.cnki.gysfc.2018.000006

[ref58] Tian Y. Song Y. Zhou Z. Huang C. Zhao Y. (2019). The development trajectory and influencing factors of teenagers' belief in a just world: a multilevel analysis. Psychol. Dev. Educ. 2, 146–156. doi: 10.16187/j.cnki.issn1001-4918.2019.02.03

[ref59] Toplu A. Çakmak S. (2024). The relationship between psychological resilience with, empathy and self-efficacy of adolescent students exposed to violence. J. Kirsehir Educ. Fac. 25, 827–862. doi: 10.29299/kefad.1237422

[ref9006] Turner M. G. Piquero A. R. Pratt T. C. (2005). The school context as a source of self-control. Journal of Criminal Justice, 33, 327–339. 10.1016/j.jcrimjus.2005.04.003

[ref9007] Turpyn C. C. Chaplin T. M. Fischer S. Thompson J. C. Fedota J. R. Baer R. A. . (2021). Affective neural mechanisms of a parenting-focused mindfulness intervention. Mindfulness, 12, 392–404. doi: 10.1007/s12671-019-01118-6 33737986 PMC7962669

[ref60] Vazsonyi A. T. Huang L. (2010). Where self-control comes from: on the development of self-control and its relationship to deviance over time. Dev. Psychol. 46, 245–257. doi: 10.1037/a0016538, 20053021

[ref61] Wallner S. Stemmler M. Reinecke J. (2021). The contributions of propensity, delinquent peers, low parental supervision, and empathy to the emergence of antisocial behavior in childhood and adolescence: testing developmental path models combining psychological-and sociological-criminological approaches. Int. J. Dev. Sci. 14, 99–112. doi: 10.3233/dev-200285

[ref62] Walters G. D. (2016). Are behavioral measures of self-control and the Grasmick selfcontrol scale measuring the same construct? A meta-analysis. Am. J. Crim. Justice 41, 151–167. doi: 10.1007/s12103-015-9317-3

[ref63] Walters G. D. (2025). Skills and competencies incompatible with crime: a theory of criminal lifestyle change. Prison J. 105, 25–43. doi: 10.1177/00328855241292789

[ref64] Wang H. Lu J. (2004). Development of a self-control questionnaire for secondary school students and its investigation. Psychology, 6, 198–203. doi: 10.16719/j.cnki.1671-6981.2004.06.055

[ref65] Wang J. Ye Y. Wang Y. Zeng X. (2024). Parenting styles and personal belief in a just world among Chinese children and adolescents: gender, living location, and age as moderators. Front. Psychol. 15:1357667. doi: 10.3389/fpsyg.2024.1357667, 39027050 PMC11256207

[ref9010] Wright J. P. Beaver K. M. (2005). Do parents matter in creating self‐control in their children? A genetically informed test of Gottfredson and Hirschi’s theory of low self‐control. Criminology, 43, 1169–1202. doi: 10.1111/j.1745-9125.2005.00036.x

[ref66] Xu Y. Y. Zhang H. Liu Y. P. (2024). The moderated mediating role of belief in a just world, family socioeconomic status, and cognitive reappraisal on adolescents' empathy. Chinese. J. Clin. Psychol. 32, 1071–1074+1079. doi: 10.16128/j.cnki.1005-3611.2024.05.022

[ref67] Zhang H. (2005). Research on the changes in juvenile justice policy in the U.K. Hebei Law Sci. 23, 2–7. doi: 10.16494/j.cnki.1002-3933.2005.02.001

[ref69] Zhou Y. Q. Gan D. Z. Q. Hoo E. C. C. Chong D. Chu C. M. (2018). Evaluating the violence prevention program: group and individual changes in aggression, anger, self-control, and empathy. J. Forensic Psychiatr. Psychol. 29, 265–287. doi: 10.1080/14789949.2017.1375541

